# Supplementary material for: Increased prevalence of transfusion-transmitted diseases among people with tattoos: A systematic review and meta-analysis
Source: PLoS One. 2022 Jan 27;17(1):e0262990. doi: 10.1371/journal.pone.0262990 (PMC8794209; doi:10.1371/journal.pone.0262990)
Supplement: S1 File — (DOCX) [file pone.0262990.s001.docx]

**Supporting information**

**S1 File. Search strategies for database.**

PubMed

(tattoo*[All Fields]) and (HIV[All Fields] or AIDS[All Fields] or immunodef*[All Fields] or hepatitis[All Fields] or HCV[All Fields] or HBV[All Fields] or HBsAg[All Fields] or syphilis[All Fields] or VDRL[All Fields] or TPHA[All Fields] or treponema*[All Fields] or transfu*[All Fields] or blood*[All Fields] or infect*[All Fields])

Web of Science

(ALL=(tattoo*) AND TS=(HIV or AIDS or immunodef* or hepatitis or HCV or HBV or HBsAg or syphilis or VDRL or TPHA or treponema* or transfu* or blood* or infect*))

Embase

tattoo* AND ('hiv'/exp OR hiv OR 'aids'/exp OR aids OR immunodef* OR 'hepatitis'/exp OR hepatitis OR hcv OR 'hbv'/exp OR hbv OR HBsAg OR 'syphilis'/exp OR syphilis OR vdrl OR tpha OR treponema* OR transfu* OR blood* OR infect*)
