# Supplementary material for: Increased prevalence of transfusion-transmitted diseases among people with tattoos: A systematic review and meta-analysis
Source: PLoS One. 2022 Jan 27;17(1):e0262990. doi: 10.1371/journal.pone.0262990 (PMC8794209; doi:10.1371/journal.pone.0262990)
Supplement: S1 Table — (DOCX) [file pone.0262990.s002.docx]

**S1 Table. Characteristics and main findings of the included studies.**

| **Study** | **Country**  **Study setting** | **Study design** | **Total (n)** | **Female**  **(n)** | **Tattooed**  **(n)** | **Non-tattooed**  **(n)** |
| --- | --- | --- | --- | --- | --- | --- |
|  |  |  |  |  |  |  |
| Azarkar et al., 2019 | Iran  Community population with tattoo | Cross-sectional study (NR^1^) | 648 | 315 | 12 | 636 |
| Hagan et al., 2019 | Georgia  General population with tattoo | Cross-sectional, nationally representative seroprevalence study | 5998 | 3868 | 626 | 5372 |
| Moradi et al., 2019 | Iran  Prisoners with tattoo | Cross-sectional biosurveillance national study | 6467 | 175 | 3105 | 3362 |
| Bielen et al., 2018 | Belgium  Patients with tattoo | Monocentric cross-sectional seroprevalence study | 2366 | 1075 | 722 | 1644 |
| Drazilova et al., 2018 | Slovakia  Community population with tattoo | Cross-sectional population-based study | 837 | 501 | 199 | 638 |
| Moradi et al., 2018 | Iran  Prisoners with tattoo | Cross-sectional biosurveillance national study | 5459 | 194 | 2362 | 3097 |
| Wasitthankasem et al., 2018 | Thailand  Community population with tattoo | Cross-sectional study (NR^1^) | 3075 | 1637 | 611 | 2464 |
| Tabasi et al., 2018 | Iran  Patients with elevated amino-transferase levels with tattoo | Cross-sectional study (NR^1^) | 150 | 54 | 37 | 113 |
| Silva et al., 2018 | Brazil  Community drug users with tattoo | Cross-sectional study | 466 | 148 | 290 | 176 |
| Poulin et al., 2018 | Canada  Prisoners with tattoo | Cross-sectional study | 1549 | 258 | 876 | 673 |
| Belaunzarán-Zamudio et al., 2017 | Mexico  All adults with tattoo imprisoned in the 10 Centers for Social Reinsertion (CERESOs) | Observational cross-sectional study nested within an HIV testing program for the General Directorate for Penal Execution and Social Re-adaptation (DGEPRS) of the Ministry of Public Safety in the State of Guanajuato in Mexico | 1919 | 163 | 819 | 1100 |
| Hodžić et al., 2017 | Zenica  Male prisoners with tattoo | Cross-sectional study | 175 | NA^2^ | 120 | 55 |
| Kebede et al., 2017 | Ethiopia  Prisoners with tattoo | Descriptive cross-sectional study | 156 | 11 | 21 | 135 |
| Wasitthankasem et al., 2017 | Thailand  Community population with tattoo | Cross-sectional study | 3036 | 1747 | 480 | 2556 |
| Silverman-Retana et al., 2017 | Mexico  Prisoners with tattoo | Cross-sectional study | 3911 | 498 | 2199 | 1712 |
| Rosińska et al., 2017 | Poland  Patients with tattoo | Cross-sectional study | 20394 | 14295 | 1247 | 19147 |
| Akhtar et al., 2016 | Pakistan  Injecting drug Users (IDUs) with tattoo | Cross-sectional study | 241 | 1 | 163 | 78 |
| Ba-Essa et al., 2016 | Saudi Arabia  Tattooed patients with diabetes mellitus | Cross-sectional study | 1057 | 717 | 19 | 1038 |
| Bhate et al., 2016 | India  Community population with tattoo | Community-based cross-sectional study | 1833 | 964 | 76 | 1757 |
| Mac Donald-Ottevanger et al., 2016 | Suriname  Patients with tattoo | Cross-sectional study (NR^1^) | 2734 | 993 | 613 | 2121 |
| Skocibusic et al., 2016 | Bosnia  IDUs with tattoo | Cross-sectional study (NR) | 120 | 60 | 71 | 49 |
| Melo et al., 2015 | Brazil  Community population with tattoo | Cross-sectional seroepidemiological study | 1001 | 607 | 68 | 933 |
| Moezzi et al., 2015 | Iran  Community population with tattoo | Cross-sectional population-based study (NR^1^) | 2958 | 1889 | 307 | 2651 |
| Nakhla et al., 2015 | Egypt  Community population with tattoo | Cross-sectional study | 2169 | 1841 | 1030 | 1139 |
| Oliveira et al., 2015 | Brazil  University employees with tattoo | Cross-sectional study (NR^1^) | 3153 | 1728 | 189 | 2964 |
| Dwibedi et al., 2014 | India  Community population with tattoo | Observational cross-sectional study | 1765 | 878 | 300 | 1465 |
| Keyvani et al., 2014 | Iran  Patients with tattoo | Cross-sectional study (NR^1^) | 6022 | 2674 | 194 | 5828 |
| Pacheco et al., 2014 | Brazil  Community drug users with tattoo | Cross-sectional study | 187 | 33 | 125 | 62 |
| Wenger et al., 2014 | USA  Prison inmates with tattoo | Cross-sectional study (NR^1^) | 304 | 55 | 97 | 207 |
| Shittu et al., 2014 | Nigeria  Blood donors with tattoo | Cross-sectional study (NR^1^) | 350 | 11 | 34 | 316 |
| Calleja-Panero et al., 2013 | Spain  Healthy working community population with tattoo | Prevalence cross-sectional study | HBV  4986  HCV  4981 | 1357 | HBV 702  HCV 652 | HBV 4284  HCV 4329 |
| Gheorghe et al., 2013 | Romania  General population in Romania with tattoo | Nationwide cross-sectional study | 12762 | 7625 | 685 | 12077 |
| Javadi et al., 2013 | Iran  IDUs with tattoo | Cross-sectional study | 539 | 28 | 293 | 246 |
| Matos et al., 2013 | Brazil  Community population with tattoo | Cross-sectional study | HBV  669  HCV  674  HIV  692  Syphilis  658 | 545 | 41 | HBV 629  HCV 634  HIV 651  Syphilis 619 |
| Navadeh et al., 2013 | Iran  Prisoners with tattoo | Cross-sectional national bio-behavioral survey | 4533 | 199 | 2041 | 2492 |
| Oliveira-Filho et al., 2013 | Brazil  Male drug users (cocaine) with tattoo | Cross-sectional study | 384 | 0 | 214 | 170 |
| Zhang et al., 2013 | China  Community female population with tattoo | Cross-sectional population-based study | 12393 | 12393 | 1626 | 10767 |
| Azevedo et al., 2012 | Brazil  Male former soccer players with tattoo | Cross-sectional study (NR^1^) | 97 | 0 | 7 | 90 |
| Ghadir et al., 2012 | Iran  Community population with tattoo | Cross-sectional study | 3656 | 1957 | 166 | 3490 |
| Hermanstyne et al., 2012 | USA  Homeless and marginally housed persons with tattoo | Cross-sectional study | 428 | 114 | 96 | 332 |
| Liakina et al., 2012 | Lithuania  National population with tattoo | Observational cross-sectional study | 399 | NA^2^ | 35 | 364 |
| Nokhodian et al., 2012 | Iran  IDUs with tattoo | Cross-sectional study | 529 | 28 | 287 | 242 |
| Strehlow et al., 2012 | USA  Community homeless population with tattoo | Community-based cross-sectional study | 387 | 105 | 147 | 240 |
| Souto et al., 2012 | Brazil  Community population with tattoo | Cross-sectional study | 3889 | 1689 | 349 | 3540 |
| Satti et al., 2012 | Pakistan  Community population with tattoo | Cross-sectional study (NR^1^) | 503 | 282 | 119 | 384 |
| Rodrigues Neto et al., 2012 | Brazil  Community population with tattoo | Epidemiological, observational,  cross-sectional study | 5013 | 3431 | 590 | 4423 |
| Abedi et al., 2011 | Iran  Healthy subjects with tattoo | Population-based cross-sectional study | 4087 | 2795 | 200 | 3887 |
| Fathimoghaddam et al., 2011 | Iran  Healthy community population with tattoo | Cross-sectional study | 1626 | 903 | 53 | 1573 |
| Jahangirnezhad et al., 2011 | Iran  Patients with tattoo | Cross-sectional study | 560 | 286 | 24 | 536 |
| Nurutdinova et al., 2011 | USA  Female substance users with tattoo | Cross-sectional study | 782 | 782 | 210 | 572 |
| Viitanen et al., 2011 | Finland  Prisoners with tattoo | Cross-sectional study (NR^1^) | 384 | 52 | 223 | 161 |
| Urbanus et al., 2011 | Netherlands  Community population with tattoo | Cross-sectional study (NR^1^) | 434 | 247 | 375 | 59 |
| Pompilio et al., 2011 | Brazil  Prisoners with tattoo | Cross-sectional study (NR^1^) | 686 | 243 | 397 | 289 |
| Lin et al., 2010 | Taiwan  Prisoners with tattoo | Cross-sectional study (NR^1^) | 12591 | 266 | 6844 | 5747 |
| Mahfoud et al., 2010 | Lebanon  Male prisoners with tattoo | Cross-sectional study (NR^1^) | 266 | NA^2^ | 163 | 103 |
| Meffre et al., 2010 | France  Patients with tattoo having a medical checkup | Cross-sectional study | 14402 | 7339 | 1053 | 13349 |
| Khin et al., 2010 | Myanmar  Blood donors with tattoo | Cross-sectional study (NR^1^) | 65236 | 8834 | 408 | 64828 |
| Teutsch et al., 2010 | Australia  Prison inmates with tattoo with a history of IDU | Hepatitis C Incidence and Transmission in Prisons Study (HITS-II) prospective cohort | 488 | 170 | 354 | 134 |
| Coelho et al., 2009 | Brazil  Prison male inmates with tattoo | Cross-sectional study (NR^1^) | 333 | NA^2^ | 120 | 213 |
| Felippe et al., 2009 | Brazil  Volunteer blood donors with tattoo | Non-randomized, transversal clinical study | 130 | NA^2^ | 37 | 93 |
| Kheirandish et al., 2009 | Iran  Male IDUs with tattoo | Cross-sectional sero-behavioral survey | 454 | NA^2^ | 125 | 329 |
| Miller et al., 2009 | Australia  IDUs with tattoo | Cross-sectional study (NR^1^) | HBV  345  HCV  361 | 252 | HBV 179  HCV 189 | HBV 166  HCV 172 |
| Zakizad et al., 2009 | Iran  Addicted male prisoners with tattoo | Cross-sectional survey | 230 | NA^2^ | 91 | 139 |
| Vickery et al., 2009 | Australia  Patients with tattoo | Cross-sectional study (NR^1^) | 2102 | 1042 | 203 | 1899 |
| Chelleng et al., 2008 | India  Community IDUs with tattoo | Cross-sectional study | 143 | 38 | 73 | 70 |
| Dandona et al., 2008 | India  Community male population with tattoo | Population- based cross-sectional study | 6230 | NA^2^ | 483 | 5747 |
| Macias et al., 2008 | Spain  Non-injecting drug users with tattoo | Cross-sectional study | 182 | 16 | 81 | 101 |
| Tavakkoli et al., 2008 | Iran  IDUs with tattoo | Cross-sectional survey | 518 | 54 | 272 | 246 |
| Sayad et al., 2008 | Iran  Community population with tattoo | Descriptive cross-sectional study | 1721 | 867 | 182 | 1539 |
| Butler et al., 2007 | Australia  Prison entrants in 7 correctional centers with tattoo | Cross-sectional study | HBV  446  HCV  450 | 58 | HBV 166  HCV 267 | HBV 180  HCV 183 |
| Lai et al., 2007 | Taiwan  Newly sentenced amphetamine-inhaling male prisoners with tattoo | Cross-sectional study | 284 | NA^2^ | 178 | 106 |
| Lim et al., 2007 | Australia  Community IDUs with tattoo | Cross-sectional sero-prevalence study | 52 | 20 | 34 | 18 |
| Mohtasham Amiri et al., 2007 | Iran  Male drug users with tattoo who are admitted to prison | Cross-sectional prevalence study | 454 | NA^2^ | 256 | 198 |
| Neumeister et al., 2007 | U.S.A.  Patients with tattoo | Cross-sectional study (NR^1^) | 243 | 161 | 145 | 98 |
| Nguyen et al., 2007 | Vietnam  Community population with tattoo | Community-based seroprevalence study (NR^1^) | 831 | 422 | 29 | 802 |
| Zamani et al., 2007 | Iran  Community drug users with tattoo | Cross-sectional study | 202 | 6 | 84 | 118 |
| Shi et al., 2007 | Taiwan  Male entering military service with tattoo | Cross-sectional study (NR^1^) | 1897 | NA^2^ | 476 | 1421 |
| Pourahmad et al., 2007 | Iran  Male prisoners with tattoo | Case-control study | HBV  1429  HCV  1431  HIV  1430 | NA^2^ | 726 | HBV 703  HCV 705  HIV 704 |
| Hwang et al., 2006 | U.S.A.  Students aged ≥18 with tattoo | Cross-sectional seroprevalence study | HBV  4321  HCV  5275 | 3283 | HBV 1108  HCV 1327 | HBV 3213  HCV 3948 |
| Jombo et al., 2006 | Nigeria  Community population with tattoo | Cross-sectional study | 300 | 107 | 101 | 199 |
| Khaja et al., 2006 | India  Male patients and volunteers from blood banks who have tattoos | Cross-sectional study (NR^1^) | 797 | NA^2^ | 70 | 727 |
| Liao et al., 2006 | Taiwan  Newly sentenced male prisoners with tattoo | Cross-sectional study | 297 | NA^2^ | 117 | 180 |
| Méndez-Sánchez et al., 2006 | Mexico  Health-care providers with tattoo | Cross-sectional study (NR^1^) | 376 | 368 | 20 | 356 |
| Sahajian et al., 2006 | France  Patients (underprivileged) with tattoo | Cross-sectional study (NR^1^) | 941 | 492 | 70 | 871 |
| Reyes et al., 2006 | Puerto Rico  IDUs with tattoo | Cross-sectional study | 331 | 89 | 241 | 90 |
| Alvarado-Esquivel et al., 2005 | Mexico  Correctional facility inmates with tattoo | Cross-sectional study | 180 | 7 | 64 | 116 |
| Babudieri et al., 2005 | Italy  Inmates in 8 prisons with tattoo | Multi-center cross-sectional study | 973 | 126 | 463 | 510 |
| Dominitz et al., 2005 | USA  Patients with tattoo | Cross-sectional population-based study | 1253 | 51 | 247 | 1006 |
| Howe et al., 2005 | USA  NIDUs with tattoo | Cross-sectional study | 708 | 212 | 265 | 443 |
| Panda et al., 2005 | India  Community male IDUs with tattoo | Cross-sectional study | 226 | NA^2^ | 98 | 128 |
| Nishioka et al., 2003 | Brazil  Patients with tattoo | Cross-sectional matched study | HBV  345  HCV  343  HIV 344  Syphilis 340 | 63 | HBV 182  HCV 180  HIV 181  Syphilis 177 | 163 |
| Ozsoy et al., 2003 | Turkey  Health-care workers with tattoo | Cross-sectional study | 702 | 302 | 29 | 673 |
| Thaisri et al., 2003 | Thailand  Male prison inmates with tattoo | Prospective cohort study | 689 | NA^2^ | 461 | 228 |
| Gani et al., 2002 | Indonesia  Drug users with tattoo | Cross-sectional study (NR^1^) | 203 | 18 | 32 | 171 |
| Gyarmathy et al., 2002 | USA  Non-injecting heroin users with tattoo | Cross-sectional study | 306 | 147 | 99 | 207 |
| Risbud et al., 2002 | India  Patients with tattoo | Cross-sectional study (NR^1^) | 497 | 111 | 194 | 303 |
| Haley et al., 2001 | USA  Patients with tattoo | Cross-sectional study | 626 | 269 | 113 | 513 |
| Müller et al., 2001 | Hungary  Blood donors with tattoo | Cross-sectional study (NR^1^) | 585 | 235 | 62 | 523 |
| Samuel et al., 2001 | USA  Community IDUs with tattoo | Cross-sectional study (NR^1^) | 773 | 276 | 577 | 196 |
| Roy et al., 2001 | Canada  Street youths with tattoo | Cross-sectional study nested Montreal Street Youth cohort study | 437 | 134 | 247 | 190 |
| Coppola et al., 2000 | Italy  Workers in contact with the public with tattoo | Cross-sectional study (NR^1^) | HBV 2950  HCV 3324 | 1875 | HBV 95  HCV 108 | HBV 2855  HCV 3216 |
| Entz et al., 2000 | Thailand  Men working on commercial fishing vessels with tattoo | Cross-sectional study | 778 | NA^2^ | 311 | 467 |
| Silverman et al., 2000 | USA  Patients with tattoo | Case-control study (NR^1^) | 212 | 105 | 106 | 106 |
| Lucas et al., 1999 | Australia  Blood donors with tattoo | Cross-sectional study (NR^1^) | 582 | 86 | 285 | 297 |
| Wada et al., 1999 | Japan  Male drug users with tattoo | Cross-sectional study | 99 | NA^2^ | 24 | 75 |
| Sawanpanyalert et al., 1996 | Thailand  Blood donors with tattoo | Cross-sectional study (NR^1^) | 1756 | 874 | 20 | 1736 |
| Rodrigues et al., 1995 | India  Patients with tattoo | Cross-sectional study (NR^1^) | 2798 | 560 | 677 | 2121 |
| Holsen et al., 1993 | Norway  New prisoners with tattoo | Cross-sectional study (NR^1^) | 70 | 2 | 30 | 40 |
| Ko et al., 1992 | Taiwan  Young men with tattoo | Case-control study | 213 | NA^2^ | 87 | 126 |
| Sebastian et al., 1992 | Brunei  Patients with tattoo | Cross-sectional study | 400 | 26 | 200 | 200 |
| Tibbs, 1987 | Kiribati  Community population with tattoo | Cross-sectional seroepidemiological study (NR^1^) | 696 | 290 | 149 | 397 |
| Hull et al., 1985 | Mexico  Male inmates with tattoo | Cross-sectional study (NR^1^) | 454 | NA^2^ | 371 | 83 |
| Olumide et al., 1976 | Nigeria  Healthy community population with tattoo | Cross-sectional study (NR^1^) | 396 | 194 | 20 | 376 |
| Patil et al., 2020 | India  Patients with tattoo | Case-control study | 158 | NA^2^ | 10 | 148 |
| Okafor et al., 2020 | Nigeria  Prison inmates with tattoo | Cross-sectional study | 142 | NA^2^ | 34 | 108 |
| Belay et al., 2020 | Ethiopia  Community population with tattoo | Cross-sectional study | 1224 | 616 | 364 | 860 |
| Mohd Suan et al., 2019 | Malaysia  Patients with tattoo | Case-control study | 510 | 138 | 36 | 474 |
| Haider et al., 2019 | Pakistan  Patients with tattoo | Case-control study | 280 | 123 | 16 | 264 |
| Moradi et al., 2020 | Iran  Prison inmates with tattoo | Cross-sectional study | 2768 | 12 | 1944 | 824 |
| Etemad et al., 2020 | Iran  Community population with tattoo | Cross-sectional study | 606 | NA^2^ | 375 | 231 |
| Shojaee et al., 2019 | Iran  Patients with tattoo | Case-control study | 3000 | 1485 | 341 | 2659 |

Abbreviations: HCV, hepatitis C virus; HBV, hepatitis B virus; HIV, human immunodeficiency virus.

^1^Not reported.

^2^Not available.
