# Supplementary material for: Increased prevalence of transfusion-transmitted diseases among people with tattoos: A systematic review and meta-analysis
Source: PLoS One. 2022 Jan 27;17(1):e0262990. doi: 10.1371/journal.pone.0262990 (PMC8794209; doi:10.1371/journal.pone.0262990)
Supplement: S2 Table — (DOCX) [file pone.0262990.s003.docx]

**S2 Table. Target diseases and related findings of the included studies.**

| **Study name** | **Target disease** | **Serological markers** | **Tattooed** | | **Non-tattooed** | |
| --- | --- | --- | --- | --- | --- | --- |
|  |  |  | **N** | **Diseased (n)** | **N** | **Diseased (n)** |
| Azarkar et al., 2019 | HBV | HBV-DNA | 12 | 3 | 636 | 58 |
| Hagan et al., 2019 | HCV | Anti-HCV | 626 | 104 | 5372 | 329 |
| Moradi et al., 2019 | HBV | HBsAg | 3105 | 101 | 3362 | 97 |
|  | HCV | Anti-HCV | 3105 | 395 | 3362 | 136 |
| Bielen et al., 2018 | HCV | Anti-HCV | 722 | 17 | 1644 | 14 |
| Drazilova et al., 2018 | HBV | HBsAg | 199 | 23 | 638 | 41 |
| Moradi et al., 2018 | HBV | HBsAg | 2362 | 60 | 3097 | 76 |
|  | HCV | Anti-HCV | 2362 | 318 | 3097 | 202 |
| Wasitthankasem et al., 2018 | HCV | Anti-HCV,  HCV RNA | 611 | 111 | 2464 | 159 |
| Tabasi et al., 2018 | HBV | HBV PCR | 37 | 10 | 113 | 18 |
|  | HCV | HCV PCR | 37 | 17 | 113 | 17 |
| Silva et al., 2018 | HCV | Anti-HCV | 290 | 108 | 176 | 24 |
| Poulin et al., 2018 | HCV | Anti-HCV | 876 | 131 | 673 | 73 |
| Belaunzarán-Zamudio et al., 2017 | HCV | Anti-HCV | 819 | 59 | 1100 | 20 |
| Hodžić et al., 2017 | HCV | Anti-HCV,  HCV RNA | 120 | 17 | 55 | 4 |
| Kebede et al., 2017 | HBV | HBsAg | 21 | 1 | 135 | 8 |
|  | HCV | Anti-HCV | 21 | 1 | 135 | 3 |
|  | HIV | HIV 1/2 Ag/Ab | 21 | 2 | 135 | 2 |
| Wasitthankasem et al., 2017 | HCV | Anti-HCV,  HCV RNA | 480 | 121 | 2556 | 183 |
| Silverman-Retana et al., 2017 | HCV | Anti-HCV | 2199 | 80 | 1712 | 23 |
| Rosińska et al., 2017 | HCV | HCV RNA | 1247 | 13 | 19147 | 86 |
| Akhtar et al., 2016 | HCV | Anti-HCV | 163 | 70 | 78 | 17 |
| Ba-Essa et al., 2016 | HCV | Anti-HCV | 19 | 2 | 1038 | 18 |
| Bhate et al., 2016 | HBV | HBsAg | 76 | 3 | 1757 | 14 |
| Mac Donald-Ottevanger et al., 2016 | HCV | Anti-HCV, HCV-RNA | 613 | 7 | 2121 | 15 |
| Skocibusic et al., 2016 | HCV | Anti-HCV | 71 | 30 | 49 | 27 |
| Melo et al., 2015 | HBV | HBsAg | 68 | 5 | 933 | 37 |
| Moezzi et al., 2015 | HCV | Anti-HCV HCV-RNA | 307 | 9 | 2651 | 17 |
| Nakhla et al., 2015 | HCV | Anti-HCV | 1030 | 103 | 1139 | 77 |
| Oliveira et al., 2015 | HCV | Anti-HCV | 189 | 4 | 2964 | 18 |
| Dwibedi et al., 2014 | HBV | HBsAg | 300 | 66 | 1465 | 203 |
| Keyvani et al., 2014 | HBV | HBsAg | 194 | 4 | 5828 | 57 |
| Pacheco et al., 2014 | HCV | Anti-HCV | 125 | 63 | 62 | 6 |
| Wenger et al., 2014 | HCV | Anti-HCV | 97 | 30 | 207 | 20 |
| Shittu et al., 2014 | HBV | HBsAg | 34 | 4 | 316 | 34 |
| Calleja-Panero et al., 2013 | HBV | HBsAg | 702 | 9 | 4284 | 27 |
|  | HCV | Anti-HCV | 652 | 9 | 4329 | 22 |
| Gheorghe et al., 2013 | HBV | HBsAg | 685 | 47 | 12077 | 507 |
| Javadi et al., 2013 | HIV | Anti-HIV | 293 | 5 | 246 | 1 |
| Matos et al., 2013 | HBV | HBsAg | 40 | 3 | 629 | 6 |
|  | HCV | Anti-HCV | 40 | 1 | 634 | 9 |
|  | HIV | HIV-1/2 EIA | 41 | 2 | 651 | 4 |
|  | Syphilis | Syphilis screening recombinant, Radim | 39 | 5 | 619 | 72 |
| Navadeh et al., 2013 | HIV | Anti-HIV1, Anti-HIV2,  HIV antigens | 2041 | 65 | 2492 | 23 |
| Oliveira-Filho et al., 2013 | HCV | HCV-RNA | 214 | 104 | 170 | 16 |
| Zhang et al., 2013 | HBV | HBsAg | 1626 | 177 | 10767 | 1002 |
| Azevedo et al., 2012 | HCV | Anti-HCV | 7 | 1 | 90 | 6 |
| Ghadir et al., 2012 | HBV | HBsAg | 166 | 6 | 3490 | 42 |
| Hermanstyne et al., 2012 | HCV | Anti-HCV | 96 | 25 | 332 | 48 |
| Liakina et al., 2012 | HCV | Anti-HCV | 35 | 8 | 364 | 21 |
| Nokhodian et al., 2012 | HCV | Anti-HCV | 287 | 158 | 242 | 91 |
| Strehlow et al., 2012 | HCV | Anti-HCV | 147 | 68 | 240 | 52 |
| Souto et al., 2012 | HCV | Anti-HCV | 349 | 39 | 3540 | 104 |
| Satti et al., 2012 | HCV | HCV RNA | 119 | 14 | 384 | 46 |
| Rodrigues Neto et al., 2012 | HCV | Anti-HCV | 590 | 4 | 4423 | 9 |
| Abedi et al., 2011 | HBV | HBsAg | 200 | 9 | 3887 | 53 |
| Fathimoghaddam et al., 2011 | HBV | HBsAg | 53 | 2 | 1573 | 21 |
| Jahangirnezhad et al., 2011 | HBV | HBsAg | 24 | 23 | 536 | 249 |
| Nurutdinova et al., 2011 | HCV | Anti-HCV | 210 | 56 | 572 | 106 |
| Viitanen et al., 2011 | HCV | Anti-HCV | 223 | 132 | 161 | 44 |
| Urbanus et al., 2011 | HBV | Anti-HBc | 375 | 17 | 59 | 1 |
| Pompilio et al., 2011 | HCV | HCV RNA | 397 | 25 | 289 | 8 |
| Lin et al., 2010 | HBV | HBsAg | 6844 | 1397 | 5747 | 737 |
| Mahfoud et al., 2010 | HCV | Anti-HCV | 163 | 9 | 103 | 0 |
| Meffre et al., 2010 | HBV | HBsAg | 1053 | 83 | 13349 | 974 |
|  | HCV | Anti-HCV | 1053 | 24 | 13349 | 93 |
| Khin et al., 2010 | HCV | Anti-HCV | 408 | 4 | 64828 | 617 |
| Teutsch et al., 2010 | HCV | Anti-HCV,  HCV RNA | 354 | 76 | 134 | 18 |
| Coelho et al., 2009 | HCV | Anti-HCV | 120 | 23 | 213 | 6 |
| Felippe et al., 2009 | HCV | Anti-HCV | 37 | 23 | 93 | 14 |
| Kheirandish et al., 2009 | HCV | Anti-HCV | 125 | 111 | 329 | 252 |
| Miller et al., 2009 | HBV | HBs Ab,  HBc Ab | 179 | 66 | 166 | 51 |
|  | HCV | Anti-HCV | 189 | 137 | 172 | 117 |
| Zakizad et al., 2009 | HCV | Anti-HCV | 91 | 54 | 139 | 28 |
| Vickery et al., 2009 | HCV | Anti-HCV | 203 | 26 | 1899 | 72 |
| Chelleng et al., 2008 | HCV | Anti-HCV | 73 | 56 | 70 | 46 |
| Dandona et al., 2008 | HIV | Anti-HIV | 483 | 27 | 5747 | 103 |
| Macias et al., 2008 | HCV | Anti-HCV, HCV-RNA | 81 | 16 | 101 | 7 |
| Tavakkoli et al., 2008 | HBV | HBsAg | 272 | 9 | 246 | 10 |
| Sayad et al., 2008 | HCV | Anti-HCV | 182 | 7 | 1539 | 8 |
| Butler et al., 2007 | HBV | HBc Ab | 266 | 61 | 180 | 27 |
|  | HCV | Anti-HCV | 267 | 112 | 183 | 44 |
| Lai et al., 2007 | HBV | HBsAg | 178 | 24 | 106 | 20 |
|  | HCV | Anti-HCV | 178 | 51 | 106 | 13 |
| Lim et al., 2007 | HCV | Anti-HCV | 34 | 23 | 18 | 6 |
| Mohtasham Amiri et al., 2007 | HCV | Anti-HCV | 256 | 136 | 198 | 70 |
| Neumeister et al., 2007 | HCV | Anti-HCV | 145 | 23 | 98 | 5 |
| Nguyen et al., 2007 | HCV | Anti-HCV | 29 | 2 | 802 | 6 |
| Zamani et al., 2007 | HCV | Anti-HCV | 84 | 49 | 118 | 56 |
| Shi et al., 2007 | HBV | HBsAg,  Anti-HBs | 476 | 54 | 1421 | 121 |
|  | HCV | Anti-HCV | 476 | 10 | 1421 | 6 |
|  | HIV | Anti-HIV 1/2 | 476 | 0 | 1421 | 0 |
|  | Syphilis | Rapid plasma reagin | 476 | 0 | 1421 | 0 |
| Pourahmad et al., 2007 | HBV | HBsAg | 726 | 30 | 703 | 16 |
|  | HCV | Anti-HCV | 726 | 337 | 705 | 160 |
|  | HIV | Anti-HIV | 726 | 73 | 704 | 19 |
| Hwang et al., 2006 | HBV | HBsAg | 1108 | 68 | 3213 | 206 |
|  | HCV | Anti-HCV | 1327 | 13 | 3948 | 35 |
| Jombo et al., 2006 | HIV | Anti-HIV 1/2 | 101 | 7 | 199 | 18 |
| Khaja et al., 2006 | HCV | Anti-HCV | 70 | 2 | 727 | 139 |
| Liao et al., 2006 | HBV | HBsAg | 117 | 21 | 180 | 23 |
|  | HCV | Anti-HCV | 117 | 17 | 180 | 13 |
| Méndez-Sánchez et al., 2006 | HBV | HBsAg  Anti-HBc | 20 | 2 | 356 | 4 |
|  | HCV | Anti-HCV | 20 | 0 | 356 | 6 |
| Sahajian et al., 2006 | HCV | Anti-HCV | 70 | 8 | 871 | 36 |
| Reyes et al., 2006 | HCV | Anti-HCV | 241 | 228 | 90 | 69 |
| Alvarado-Esquivel et al., 2005 | HCV | Anti-HCV | 64 | 17 | 116 | 1 |
| Babudieri et al., 2005 | HBV | HBc Ab | 463 | 252 | 510 | 260 |
|  | HCV | Anti-HCV | 463 | 237 | 510 | 133 |
|  | HIV | HIV  ELISA | 463 | 55 | 510 | 18 |
| Dominitz et al., 2005 | HCV | Anti-HCV | 247 | 28 | 1006 | 22 |
| Howe et al., 2005 | HCV | Anti-HCV | 265 | 11 | 443 | 15 |
| Panda et al., 2005 | HIV | Anti-HIV | 98 | 62 | 128 | 6 |
| Nishioka et al., 2003 | HBV | HBsAg HBcAb | 182 | 39 | 163 | 14 |
|  | HCV | Anti-HCV | 180 | 32 | 163 | 2 |
|  | HIV | Anti-HIV | 181 | 28 | 163 | 1 |
|  | Syphilis | VDRL | 177 | 12 | 163 | 5 |
| Ozsoy et al., 2003 | HBV | HBsAg | 29 | 1 | 673 | 20 |
| Thaisri et al., 2003 | HIV | Anti-HIV | 461 | 158 | 228 | 17 |
| Gani et al., 2002 | HBV | HBsAg | 32 | 2 | 171 | 17 |
|  | HCV | Anti-HCV | 32 | 31 | 171 | 120 |
| Gyarmathy et al., 2002 | HBV | Anti-HBc | 99 | 24 | 207 | 45 |
|  | HCV | Anti-HCV | 99 | 18 | 207 | 19 |
|  | HIV | Anti-HIV-1 | 99 | 6 | 207 | 22 |
| Risbud et al., 2002 | HBV | Anti-HBc | 194 | 111 | 303 | 104 |
| Haley et al., 2001 | HCV | Anti-HCV | 113 | 25 | 513 | 18 |
| Müller et al., 2001 | HCV | Anti-HCV | 62 | 42 | 523 | 153 |
| Samuel et al., 2001 | HBV | Anti-HBc HBsAg | 577 | 368 | 196 | 100 |
|  | HCV | Anti-HCV | 577 | 189 | 196 | 143 |
| Roy et al., 2001 | HCV | Anti-HCV | 247 | 45 | 190 | 10 |
| Coppola et al., 2000 | HBV | HBsAg, HBcAb | 95 | 33 | 2855 | 794 |
|  | HCV | Anti-HCV | 108 | 18 | 3216 | 62 |
| Entz et al., 2000 | HIV | HIV IgG | 311 | 68 | 467 | 53 |
| Silverman et al., 2000 | HBV | Anti-HBc  HBV DNA | 106 | 1 | 106 | 0 |
|  | HCV | Anti-HCV HCV RNA | 106 | 7 | 106 | 3 |
| Lucas et al., 1999 | HCV | Anti-HCV | 285 | 21 | 297 | 14 |
| Wada et al., 1999 | HBV | HBsAg | 24 | 1 | 75 | 2 |
|  | HCV | Anti-HCV | 24 | 15 | 75 | 18 |
|  | HIV | Anti-HIV | 24 | 0 | 75 | 0 |
| Sawanpanyalert et al., 1996 | HCV | Anti-HCV | 20 | 2 | 1736 | 37 |
|  | HIV | Anti-HIV | 20 | 1 | 1736 | 48 |
| Rodrigues et al., 1995 | HIV | Anti-HIV-1 | 677 | 191 | 2121 | 452 |
| Holsen et al., 1993 | HCV | Anti-HCV | 30 | 22 | 40 | 10 |
| Ko et al., 1992 | HCV | Anti-HCV | 87 | 11 | 126 | 3 |
| Sebastian et al., 1992 | HBV | HBsAg | 200 | 39 | 200 | 18 |
| Tibbs, 1987 | HBV | HBsAg | 299 | 49 | 397 | 118 |
| Hull et al., 1985 | HBV | HBsAg  Anti-HBs  Anti-HBc | 371 | 187 | 83 | 26 |
| Olumide et al., 1976 | HBV | HBsAg | 20 | 1 | 376 | 49 |
| Patil et al., 2020 | HIV | Anti-HIV | 10 | 4 | 148 | 29 |
| Okafor et al., 2020 | HCV | Anti-HCV | 34 | 8 | 108 | 34 |
| Belay et al., 2020 | HBV | HBsAg | 364 | 68 | 860 | 42 |
| Mohd Suan et al., 2019 | HCV | Anti-HCV,  HCV RNA | 36 | 31 | 474 | 224 |
| Haider et al., 2019 | HIV | Anti-HIV-1 | 16 | 14 | 264 | 126 |
| Moradi et al., 2020 | HBV | HBsAg | 972 | 25 | 412 | 11 |
|  | HCV | Anti-HCV | 972 | 442 | 412 | 146 |
| Etemad et al., 2020 | HIV | Anti-HIV | 375 | 34 | 231 | 5 |
| Shojaee et al., 2019 | HBV | HBsAg | 341 | 167 | 2659 | 1833 |

Abbreviations: HCV, hepatitis C virus; HBV, hepatitis B virus; HIV, human immunodeficiency virus.
