# Supplementary material for: Increased prevalence of transfusion-transmitted diseases among people with tattoos: A systematic review and meta-analysis
Source: PLoS One. 2022 Jan 27;17(1):e0262990. doi: 10.1371/journal.pone.0262990 (PMC8794209; doi:10.1371/journal.pone.0262990)
Supplement: S3 Table — (DOCX) [file pone.0262990.s004.docx]

**S3 Table. Quality assessment of analytical studies that used the Adapted Newcastle-Ottawa Scale for Cross-Sectional Studies.**

| **Study name** | **Sample represent ability (random sampling/whole population)** | **Adequate sample size (>300)** | **Exposure ascertainment** | **Non-response rate** | **Matching for age/sex** | **Additional matching** | **Outcome assessment** | **Adequate statistical test** | **Overall quality score (max 8)** |
| --- | --- | --- | --- | --- | --- | --- | --- | --- | --- |
| Azarkar et al., 2019 | a | a | a | b | - | - | a | a | 5 |
| Hagan et al., 2019 | a | a | a | b | - | - | a | a | 5 |
| Moradi et al., 2019 | a | a | a | b | - | - | a | a | 6 |
| Bielen et al., 2018 | b | a | a | b | - | - | a | a | 4 |
| Drazilova et al., 2018 | a | a | a | b | - | - | a | a | 5 |
| Moradi et al., 2018 | a | a | a | a | - | - | a | a | 6 |
| Wasitthankasem et al., 2018 | a | a | a | b | - | - | a | a | 5 |
| Tabasi et al., 2018 | c | b | a | b | - | - | a | a | 3 |
| Silva et al., 2018 | b | a | a | c | - | - | a | a | 4 |
| Poulin et al., 2018 | b | a | a | b | - | - | a | a | 4 |
| Belaunzarán-Zamudio et al., 2017 | a | a | a | a | - | - | a | a | 6 |
| Hodžić et al., 2017 | b | a | a | b | - | - | a | a | 4 |
| Kebede et al., 2017 | a | b | a | a | - | - | a | a | 5 |
| Wasitthankasem et al., 2017 | a | a | a | a | - | - | a | a | 6 |
| Silverman-Retana et al., 2017 | a | a | a | a | - | - | a | a | 6 |
| Rosińska et al., 2017 | a | a | a | b | - | - | a | a, | 5 |
| Akhtar et al., 2016 | c | b | a | c | - | - | a | a | 3 |
| Ba-Essa et al., 2016 | a | a | a | a | - | - | a | a | 6 |
| Bhate et al., 2016 | a | a | a | b | - | - | a | a | 5 |
| Mac Donald-Ottevanger et al., 2016 | b | a | a | b | - | - | a | a | 4 |
| Skocibusic et al., 2016 | a | b | a | a | - | - | a | a | 5 |
| Melo et al., 2015 | b | a | a | c | - | - | a | a | 4 |
| Moezzi et al., 2015 | b | a | a | b | - | - | a | a | 4 |
| Nakhla et al., 2015 | a | a | a | a | - | - | a | a | 6 |
| Oliveira et al., 2015 | b | a | a | b | - | - | a | a | 4 |
| Dwibedi et al., 2014 | b | a | a | b | - | - | a | a | 4 |
| Keyvani et al., 2014 | a | a | a | a | - | - | a | a | 6 |
| Pacheco et al., 2014 | c | b | a | b | - | - | a | a | 3 |
| Wenger et al., 2014 | b | a | a | b | - | - | a | a | 4 |
| Shittu et al., 2014 | b | a | a | c | - | - | a | a | 4 |
| Calleja-Panero et al., 2013 | b | a | a | b | - | - | a | a | 4 |
| Gheorghe et al., 2013 | a | a | a | b | - | - | a | a | 5 |
| Javadi et al., 2013 | b | a | a | c | - | - | a | a | 4 |
| Matos et al., 2013 | b | a | a | b | - | - | a | a | 4 |
| Navadeh et al., 2013 | a | a | a | b | - | - | a | a | 5 |
| Oliveira-Filho et al., 2013 | a | b | a | a | - | - | a | a | 5 |
| Zhang et al., 2013 | a | a | a | b | - | - | a | a | 5 |
| Azevedo et al., 2012 | c | b | a | a | - | - | a | a | 4 |
| Ghadir et al., 2012 | b | a | a | b | - | - | a | a | 4 |
| Hermanstyne et al., 2012 | b | a | a | b | - | - | a | a | 4 |
| Liakina et al., 2012 | a | a | a | b | - | - | a | a | 5 |
| Nokhodian et al., 2012 | b | a | a | b | - | - | a | a | 4 |
| Strehlow et al., 2012 | b | a | a | b | - | - | a | a | 4 |
| Souto et al., 2012 | a | a | a | c | - | - | a | a | 5 |
| Satti et al., 2012 | a | a | a | a | - | - | a | a | 6 |
| Rodrigues Neto et al., 2012 | a | a | a | c | - | - | a | a | 5 |
| Abedi et al., 2011 | a | a | a | a | - | - | a | a | 6 |
| Fathimoghaddam et al., 2011 | a | a | a | b | - | - | a | a | 5 |
| Jahangirnezhad et al., 2011 | c | a | a | c | - | - | a | a | 4 |
| Nurutdinova et al., 2011 | a | a | a | c | - | - | a | a | 5 |
| Viitanen et al., 2011 | a | a | a | b | - | Sociodemographic and criminological backgrounds | a | a | 6 |
| Urbanus et al., 2011 | b | a | a | c | - | - | a | a | 4 |
| Pompilio et al., 2011 | b | a | a | c | - | - | a | a | 4 |
| Lin et al., 2010 | b | a | a | b | - | - | a | a | 4 |
| Mahfoud et al., 2010 | a | b | a | c | - | - | a | a | 4 |
| Meffre et al., 2010 | a | a | a | a | - | - | a | a | 6 |
| Khin et al., 2010 | b | a | a | c | - | - | a | a | 4 |
| Teutsch et al., 2010 | a | a | a | c | - | - | a | a | 5 |
| Coelho et al., 2009 | a | a | a | b | - | - | a | a | 5 |
| Felippe et al., 2009 | b | b | a | c | - | - | a | a | 3 |
| Kheirandish et al., 2009 | b | a | a | b | - | - | a | a | 4 |
| Miller et al., 2009 | b | a | a | c | - | - | a | a | 4 |
| Zakizad et al., 2009 | a | a | a | a | - | - | a | a | 6 |
| Vickery et al., 2009 | c | a | a | b | - | - | a | a | 4 |
| Chelleng et al., 2008 | a | b | a | c | - | - | a | a | 4 |
| Dandona et al., 2008 | a | a | a | b | - | - | a | a | 5 |
| Macias et al., 2008 | b | b | a | c | - | - | a | a | 3 |
| Tavakkoli et al., 2008 | c | a | a | c | - | - | a | a | 4 |
| Sayad et al., 2008 | b | a | a | b | - | - | a | a | 4 |
| Butler et al., 2007 | b | a | a | b | - | - | a | a | 4 |
| Lai et al., 2007 | b | b | a | a | - | - | a | a | 4 |
| Lim et al., 2007 | b | b | a | c | - | - | a | a | 3 |
| Mohtasham Amiri et al., 2007 | b | a | a | a | - | - | a | a | 5 |
| Neumeister et al., 2007 | c | b | a | b | - | - | a | a | 3 |
| Nguyen et al., 2007 | a | a | a | b | - | - | a | a | 5 |
| Zamani et al., 2007 | b | a | a | b | - | - | a | a | 4 |
| Shi et al., 2007 | b | a | a | c | - | - | a | a | 4 |
| Pourahmad et al., 2007 | b | a | a | c | - | - | a | a | 4 |
| Hwang et al., 2006 | b | a | a | c | - | - | a | a | 4 |
| Jombo et al., 2006 | a | a | a | c | - | - | a | a | 4 |
| Khaja et al., 2006 | b | a | a | c | - | - | a | a | 4 |
| Liao et al., 2006 | a | b | a | a | - | - | a | a | 5 |
| Méndez-Sánchez et al., 2006 | b | a | a | b | - | - | a | a | 4 |
| Sahajian et al., 2006 | a | a | a | a | - | - | a | a | 6 |
| Reyes et al., 2006 | a | a | a | c | - | - | a | a | 5 |
| Alvarado-Esquivel et al., 2005 | a | b | a | c | - | - | a | a | 4 |
| Babudieri et al., 2005 | a | a | a | a | - | - | a | a | 6 |
| Dominitz et al., 2005 | a | a | a | a | - | - | a | a | 6 |
| Howe et al., 2005 | b | a | a | a | - | - | a | a | 5 |
| Panda et al., 2005 | b | b | a | b | - | - | a | a | 3 |
| Nishioka et al., 2003 | b | a | a | b | Age/sex | Main clinical complaint | a | a | 6 |
| Ozsoy et al., 2003 | c | a | a | c | - | - | a | a | 4 |
| Thaisri et al., 2003 | b | a | a | c | - | - | a | a | 4 |
| Gani et al., 2002 | b | b | a | c | - | - | a | a | 3 |
| Gyarmathy et al., 2002 | b | a | a | b | - | - | a | a | 4 |
| Risbud et al., 2002 | a | a | a | c | - | - | a | a | 5 |
| Haley et al., 2001 | b | a | a | c | - | - | a | a | 4 |
| Müller et al., 2001 | a | a | a | c | - | - | a | a | 5 |
| Samuel et al., 2001 | b | a | a | b | - | - | a | a | 4 |
| Roy et al., 2001 | b | a | a | c | - | - | a | a | 4 |
| Coppola et al., 2000 | a | a | a | b | - | - | a | a | 5 |
| Entz et al., 2000 | b | a | a | c | - | - | a | a | 4 |
| Silverman et al., 2000 | c | b | a | c | - | - | a | a | 3 |
| Lucas et al., 1999 | b | a | a | c | - | - | a | a | 4 |
| Wada et al., 1999 | c | b | a | b | - | - | a | a | 3 |
| Sawanpanyalert et al., 1996 | b | a | a | a | - | - | a | a | 5 |
| Rodrigues et al., 1995 | b | b | a | b | - | - | a | a | 3 |
| Holsen et al., 1993 | b | b | a | b | - | - | a | a | 3 |
| Ko et al., 1992 | b | b | a | c | Age/sex | Education, occupation, geographic origin | a | a | 5 |
| Sebastian et al., 1992 | a | a | a | c | Age/sex | Ethnic group | a | a | 7 |
| Tibbs, 1987 | b | a | a | c | - | - | a | a | 4 |
| Hull et al., 1985 | b | a | a | b | - | - | a | a | 4 |
| Olumide et al., 1976 | b | a | a | c | - | - | a | a | 4 |
| Patil et al., 2020 | b | b | a | c | - | - | a | a | 3 |
| Okafor et al., 2020 | a | b | a | c | - | - | a | a | 4 |
| Belay et al., 2020 | a | a | a | b | - | - | a | a | 4 |
| Mohd Suan et al., 2019 | b | a | a | c | Age/sex | Ethnicity | a | a | 6 |
| Haider et al., 2019 | a | b | a | c | - | - | a | a | 4 |
| Moradi et al., 2020 | a | a | a | c | - | - | a | a | 5 |
| Etemad et al., 2020 | b | a | a | c | - | - | a | a | 4 |
| Shojaee et al., 2019 | b | a | a | b | - | - | a | a | 4 |
