# Supplementary material for: Increased prevalence of transfusion-transmitted diseases among people with tattoos: A systematic review and meta-analysis
Source: PLoS One. 2022 Jan 27;17(1):e0262990. doi: 10.1371/journal.pone.0262990 (PMC8794209; doi:10.1371/journal.pone.0262990)
Supplement: Attachment — Submitted filename: Response_to_Reviewers.docx [file pone.0262990.s006.docx]

Dear editors and reviewers,

We appreciate your valuable feedback. We have revised our manuscript in accordance with the reviewers’ comments. Our responses to these comments are presented below.

Journal Requirements:

When submitting your revision, we need you to address these additional requirements.

1. Please ensure that your manuscript meets PLOS ONE's style requirements, including those for file naming. The PLOS ONE style templates can be found at

https://journals.plos.org/plosone/s/file?id=wjVg/PLOSOne_formatting_sample_main_body.pdf and

https://journals.plos.org/plosone/s/file?id=ba62/PLOSOne_formatting_sample_title_authors_affiliations.pdf

2. Please review your reference list to ensure that it is complete and correct. If you have cited papers that have been retracted, please include the rationale for doing so in the manuscript text, or remove these references and replace them with relevant current references. Any changes to the reference list should be mentioned in the rebuttal letter that accompanies your revised manuscript. If you need to cite a retracted article, indicate the article’s retracted status in the References list and also include a citation and full reference for the retraction notice.

 Response:

As recommended, we have updated our manuscript according to the journal’s style, including the file name and the overall format of the manuscript.

We have also amended the references. We removed reference #16, which has been deleted from the journal’s website. We then thoroughly reviewed our cited literature and found no retracted articles.

3. During our internal review, we noticed you have overlapping text with an abstract of your work published here:

http://www.papersearch.net/thesis/article.asp?key=3845077

PLOS ONE cannot (re)publish material without sufficient permission from the original copyright holder to publish under a CC BY license. Please provide proof that the owner of the content (a) has given you written permission to use it, and (b) has approved of the CC BY license being applied to their content. You may have the following form completed by the owner as proof: https://journals.plos.org/plosone/s/file?id=7c09/content-permission-form.pdf. Alternatively, you may electronically request permissions electronically from the copyright owner and send us proof of approval, as long as the approval clearly shows that the owner has approved of the CC BY license being applied to their content. Please see https://journals.plos.org/plosone/s/licenses-and-copyright for more information.

Response:

Thank you for your careful review of our manuscript.

The study mentioned on the papersearch website is the same as ours. We had previously presented our study (abstract only) in the Korean Dermatologists’ Annual Autumn meeting as a conference proceeding. We have attached the proof of approval from the Korean Dermatological Association.

**5. Review Comments to the Author**
Reviewer #1: This article appears to be a rigorously conducted literature review. You accessed observational data comparing tattooed populations to non-tattooed populations to determine prevalence of Hepatitis B, C, HIV and syphilis.

**However, a few of the statements in your conclusion suggest causation, and you have not been able to determine causation via this literature review, only association.** For example, on page 12, lines 202-206, you state that: "the incidence of TTDs in the tattooed population is higher than in the general population BECAUSE having or performing tattoos is a modifiable factor for preventing the spread of such diseases...our study indicates an INCREASE IN THE RISK OF TTDs among subjects with tattoos." Finally, at the end of the Discussion section, page 15, lines 274-275, you state: "tattooing procedures performed under unhygienic conditions SEEM TO BE THE CORE MATTER IN THE SPREAD OF TTDs."

I interpret these statements as indicating that tattooing is responsible for the spread of TTDs, but I don't believe the is what your data show. Instead, they show that the likelihood of having a TTD is higher among those with tattoos, but there is no causal evidence in your review. The authors try to address this by doing analyses with different sub-populations, such as prisoners and IV drug users.

**However, there is no statement in the article that addresses the possibility that other behaviors that may be more common among people with tattoos** could be responsible for the spread of TTDs and there was no discussion of the conditions under which the people in these studies were tattooed (ie, there is a huge difference between getting a tattooed from a licensed artist who has received training on prevention of communicable diseases and an untrained person in a prison who is not using sterilized, single-use equipment). **In addition, there is no discussion of the timing of the TTD transmission and the tattoo**. For these reasons, that statements mentioned above should be reworded to be clear that there was no proof of causation, but only association**. I do not see a limitations section either where you could acknowledge that there could be other behaviors that predisposed these individuals to TTDs.**

**Additionally, it would be useful if you define the term "community-dweller" as it is not immediately clear to the reader who is being described by this term.**

Response:

We greatly appreciate your suggestions regarding our manuscript. We have carefully reviewed and updated the text as suggested.

First, we have revised all the sentences that stated or implied a causal relationship between tattooing and TTD infections. The sentence you first pointed out has been deleted. The expressions on page 12, lines 210-212; page 14, lines 242-245; page 16, lines 290-292, have been revised into objective phrases that interpret the results of the data (highlighted in the revised manuscript).

- Page 12, lines 210-212:

In line with previous studies, the results of our study show that the likelihood of having TTDs (HCV, HBV, and HIV infections) among subjects with tattoos is higher than that of the non-tattooed population.

- Page 14, lines 242-245:

Overall, although our study did not differentiate prison inmates with tattoos before and after the incarceration, the results imply that prisoners and IV drug users who might have been exposed to an unsafe environment during tattooing could contribute to the increased prevalence of TTDs.

- Page 16, lines 290-292:

Apart from the hazardous effects of the tattoo materials themselves, the unhygienic conditions in which the procedures are performed may be associated with the spread of TTDs.

Second, we strongly agree with your opinion that other factors may influence the incidence of TTDs. Thus, we have addressed possible confounding factors in the revised manuscript.

A study has scrutinized tattoo-associated behaviors. Drews et al. [1] investigated behavioral differences in tattooed and non-tattooed college students using self-evaluation questionnaires. The tattooed male students’ responses showed increased incidence of participation in risky behaviors, presence of more sexual partners, and higher arrest rates. The responses of tattooed females revealed an increased incidence of drug use and body piercings. These behaviors may also have been associated with the increased prevalence of TTDs.

In addition, establishing a temporal relationship between having a tattoo and the morbidity of the TTDs is also essential; however, we could not clarify this relationship owing to the lack of information in the included studies.

We have added the underlined explanation in the Discussion section and also stated this point as a limitation in our revised manuscript (highlighted, page 15, lines 268-274 and lines 280-285). The limitation section is within discussion section, page 15, lines 275-285.

Lastly, we defined “community-dwellers” as those who live within certain geographic areas, in accordance with the studies included (page 7, lines 124-128).

1. Drews DR, Allison CK, Probst JR. Behavioral and self-concept differences in tattooed and nontattooed college students. Psychol Rep. 2000; 86(2):475-81. https://doi.org/10.2466/pr0.2000.86.2.475 PMID:10840898.
