# Supplementary material for: Increased prevalence of transfusion-transmitted diseases among people with tattoos: A systematic review and meta-analysis
Source: PLoS One. 2022 Jan 27;17(1):e0262990. doi: 10.1371/journal.pone.0262990 (PMC8794209; doi:10.1371/journal.pone.0262990)
Supplement: Attachment — Submitted filename: Response_to_Reviewers_Final.docx [file pone.0262990.s007.docx]

**PONE-D-21-05095R2**

**Increased prevalence of transfusion-transmitted diseases among people with tattoos: A systematic review and meta-analysis**

PLOS ONE

Reviewers' comments:

**Reviewer #1:**

Thank you for your revisions. You have addressed my comments sufficiently, except that the term "community dweller" is still unclear to me. I believe perhaps you are referring to people who live in urban areas? In which case, you could put urban dwellers or city dwellers? Otherwise, it remains unclear why someone who lives within a certain geographic area would be at higher risk for TTD's, unless you describe some other particular characteristics of those geographic areas.

Response:

Thank you for your considerate comment.

The term “community-dwellers” was initially defined as people who live within certain geographic areas (urban as well as rural) according to **population-based studies**. However, we absolutely agree with your comment that this term is vague.

We believe that this community subgroup referred to in population studies could represent the **general population,** apart from the specific populations and circumstances to which they belong. Other subgroups defined in our study included hospitalized patients, blood donors, intravenous drug users, and prisoners. Such subgroups are within characteristic environments which favor an increased prevalence of TTDs in the tattooed population. **Thus, we thought we could evaluate general prevalence by assessing community-based subgroup.** Certainly, the community sample was not evenly recruited from all over the world; however, **we thought it is worth estimating the prevalence in a sample which can be approximated to the general population.**

To convey the meaning of the term properly, we revised the term “community-dwellers” to “**general population**”.

We added such descriptions and replaced the original term with “general population” in the revised manuscript (Highlighted, lines 126-130)
